# The Initiative to Maximize Progress in Adolescent and Young Adult Cancer Therapy (IMPACT) Cohort Study: a population-based cohort of young Canadians with cancer

**DOI:** 10.1186/1471-2407-14-805

**Published:** 2014-11-03

**Authors:** Nancy N Baxter, Corinne Daly, Sumit Gupta, Jason D Pole, Rinku Sutradhar, Mark L Greenberg, Paul C Nathan

**Affiliations:** Department of Surgery, St. Michael’s Hospital, 30 Bond Street, Toronto, ON M5B 1W8 Canada; Kennan Research Centre, St. Michael’s Hospital, Toronto, Canada; Institute for Clinical Evaluative Sciences, Toronto, Canada; Institute of Health Policy, Management and Evaluation, University of Toronto, Toronto, Canada; The Hospital for Sick Children, Toronto, Canada; Dalla Lana School of Public Health, University of Toronto, Toronto, Canada; Pediatric Oncology Group of Ontario, Toronto, Canada

**Keywords:** Adolescents, Young adults, Cancer, Treatment, Recurrence, Survival, Cohort, Population-based

## Abstract

**Background:**

Cancer is the leading cause of disease-related death in adolescents and young adults (AYA). Annual improvements in AYA cancer survival have been inferior to those observed in children and older adults. Prior studies of AYA with cancer have been limited by their focus on patients from select treatment centres, reducing generalizability, or by being population-based but lacking diagnostic and treatment details. There is a critical need to conduct population-based studies that capture detailed patient, disease, treatment and system-level data on all AYA regardless of treatment location.

**Methods/Design:**

We will create a cohort of all AYA (aged 15–21 years) at the time of diagnosis with any malignancy between 1992 and 2011 in Ontario, Canada (n = 5,394). Subjects will be identified through the Ontario Cancer Registry and the final cohort will be expanded to include 2012 diagnoses, as these data become available. Detailed diagnostic, treatment and outcome data for those patients treated at a pediatric cancer centre will be provided by a population-based pediatric cancer registry (n = 1,030). For 15–18 year olds treated at adult centres (n = 923) and all 19–21 year olds (n = 3396), trained abstractors will collect the comparable data elements from medical records. We will link these data to population-based administrative health data that include physician billings, hospitalizations and emergency room visits. This will allow descriptions of health care access and use prior to cancer diagnosis, and during and after treatment.

**Discussion:**

The IMPACT cohort will serve as a platform for addressing questions that span the AYA cancer journey. These will include determining which factors influence where AYA receive care, the impact of locus of care on the types and intensity of cancer therapy, appropriateness of surveillance for disease recurrence, access to clinical trials, and receipt of palliative and survivor care. Findings using the IMPACT cohort have the potential to lead to changes in practice and cancer policy, reduce mortality, and improve quality of life for AYA with cancer. The IMPACT data platform will be a permanent resource, accessible to researchers across Canada.

## Background

Over 2,300 Canadians aged 15–29 years develop cancer annually [1]. Cancer is the leading cause of disease-related death in adolescents and young adults (AYA) [2–5], yet improvements in survival and focused research lag behind that in children and older adults. Over the past 25 years, annual improvement in 5-year cancer survival has exceeded 1.5% in both children <15 years and adults >50 years [6]. In contrast, the annual improvement in survival has been less than 0.5% in 15–24 year olds and non-existent in those aged 25–29 [3, 6–8]. The reasons for the disparities are unclear, but likely include patient, disease, and health care system factors including unfavorable tumour biology [9, 10], increased risk for acute toxicity from therapy [11–14], poor adherence to therapy [15], vulnerability to diagnostic delay [16–18] resulting in advanced stage at diagnosis [18, 19] and limited opportunities to participate in clinical trials [6, 20].

Location of cancer therapy may exacerbate or mitigate the above vulnerabilities. In Ontario, 19% of 15–21 year olds are treated at a pediatric cancer centre, 57% at an adult Regional Cancer Centre (RCC), and 24% at a community hospital (unpublished data). Most care settings do not have specific programs focused on addressing the differences in disease biology and response to therapy [20–22] or the risks for toxicity and late effects of therapy including infertility [23–25] cardiac, pulmonary or other treatment repercussions [26–28], secondary malignancies [29, 30], as well as the unique health and psychosocial issues faced by AYA, such as difficulty reentering school or the workforce, and forming or maintaining romantic relationships [31–33]. Despite recommendations that AYA cancer therapy be administered by experts in AYA oncology [34], AYA comprise a small percentage of patients seen in either pediatric or adult centres [2, 34]. This results in a paucity of AYA expertise, which may lead to variations in care and treatment intensity between sites. AYA who receive their therapy in a community hospital are particularly vulnerable; in a recent analysis of Ontario AYA with lymphoma, we demonstrated that those patients who were treated in a cancer centre (pediatric or adult) had a 16% higher likelihood of survival than those treated in a community hospital [35]. Disparities in outcome by LOC have been observed in leukemia, sarcoma, non-Hodgkin lymphoma (NHL) and brain tumours [36–40].

Disparities in AYA cancer care and outcomes extend beyond survival to encompass end-of-life and survivor care. Studies of survivors of childhood cancer (including some AYA) have found that the majority will develop late effects of therapy that are often severe and can lead to premature death [41–44]. Since adolescence and young adulthood is a period of substantial physical and emotional development, AYA may be particularly vulnerable to these late effects. Variations in care according to LOC may impact outcomes in AYA cancer survivors. For example, treatment of Hodgkin’s Lymphoma with adult-type anthracycline-based therapy increases the risk for cardiac disease, while pediatric regimens that contain alkylating agents may have greater impact on fertility [45–47].

Although LOC likely impacts cancer survival, risk for late effects, and access to palliative and survivor care, little is known about determinants of LOC in AYA. A US study found that younger age and cancer type influenced the chance of AYA being referred to a pediatric centre [48]. Cancers such as ALL were more likely to be treated in a pediatric centre; thyroid and other carcinomas were more frequently treated at an adult centre. An AYA’s primary care practitioner’s (PCP) specialty likely influences referral patterns, but this variable has not been studied. The patient, disease and system factors that determine LOC in Canada’s universal health care system have not been examined.

Here we report on the design and methods of the Initiative to Maximize Progress in AYA Cancer Therapy (IMPACT) Study. This is the first population-based cohort study of all AYA aged 15–21 with complete diagnostic, treatment, and outcomes data. The IMPACT study will address several gaps in the literature. We aim to:

1. Determine patient and healthcare system factors that determine LOC;
2. Identify whether LOC is associated with variation in care across the cancer continuum, including intensity and type of cancer therapy, clinical trial enrollment, guideline-recommended survivor care and end-of-life palliation;
3. Examine the relationship between LOC and survival within malignancy groups accounting for potential confounders (patient demographics, stage at diagnosis, disease biology). For those malignancies in which overall or event-free survival differs by LOC, to determine the impact of the variations in care on survival disparities.

## Methods

### Overview

**Figure 1:**
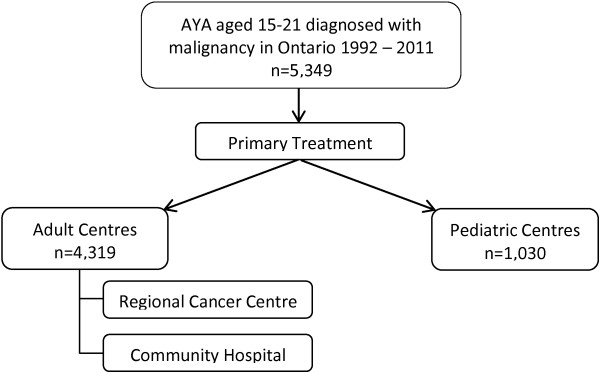
**Eligibility for the IMPACT cohort.**

**Figure 2:**
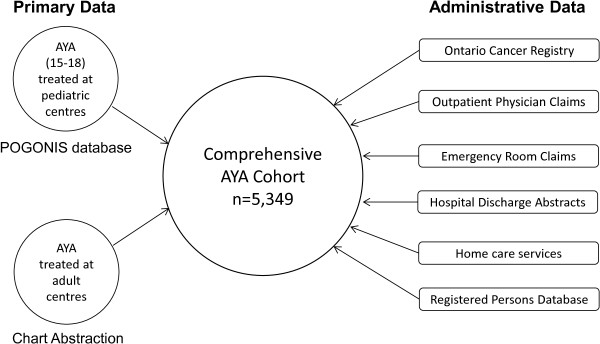
**Primary data on members of IMPACT cohort will be linked to multiple administrative datasets held at the Institute for Clinical Evaluative Sciences to create a comprehensive cohort of all AYA in Ontario including demographic, diagnosis, treatment, recurrence, outcomes and health services use information.** Abbreviation: POGONIS, Pediatric Oncology Group of Ontario Networked Information System.

This study will include 5,349 AYA aged 15–21 years diagnosed with any malignancy in the Ontario from 1992–2011 (Figure 1). The cohort will be identified through the Ontario Cancer Registry (OCR) and expanded to include diagnoses in 2012, as these data become available. The OCR, operated by Cancer Care Ontario, captures information on all incident cancers in Ontario since 1964 and is over 95% complete [49]. Data on disease characteristics, treatment and outcomes will be collected using linkage to the Pediatric Oncology Group of Ontario Networked Information System (POGONIS) for patients treated at pediatric centres and rigorous chart abstraction for patients treated elsewhere. The number of included AYA by LOC for common malignancies is estimated in Table 1. Linking this cohort to health services and other population-based databases housed at the Institute for Clinical Evaluative Sciences (ICES) will enable collection of data regarding demographics, healthcare utilization before, during and after treatment, and key short- and long-term outcomes (Figure 2). These databases are not publicly accessible. Permission was granted to access POGONIS by the Pediatric Oncology Group of Ontario and remaining databases are accessible upon approval of the privacy office at ICES. ICES and POGO are named prescribed entities under section 45(1) of Ontario’s *Personal Health Information Protection Act (PHIPA, 2004).* This permits health information custodians (such as hospitals) to disclose PHI to these agencies “*for the purpose of analyzing and/or compiling statistical information with respect to the management of, evaluation or monitoring of, the allocation of resources to or planning for all or part of the health system, including the delivery of services*” without individual consent. The Research Ethics Board at St. Michael’s Hospital, Toronto, Canada has approved the protocol for this study (12–233).

**Table 1:** **Distribution of locus of care for the most common adolescent and young adult malignancies, ages 15–21, in Ontario, Canada**

			Adult n (%)	
Malignancy type	N	Pediatric n (%)	RCC	Community
Hodgkin lymphoma	986	238 (24.1)	710 (72.0)	38 (3.9)
Thyroid cancer	677	34 (5.1)	227(33.5)	415 (61.4)
Bone/soft tissue sarcomas	545	152 (27.9)	273 (50.1)	120 (22.0)
Testicular cancer	507	30 (6.0)	422 (83.3)	54 (10.7)
Leukemia	483	184 (38.0)	255 (52.7)	45 (9.3)
Brain tumours	482	172 (25.7)	221 (45.8)	89 (18.5)
Non-Hodgkin lymphoma	421	109 (25.8)	212 (50.4)	100 (23.8)
Other	1248	241 (19.3)	715 (57.3)	292 (23.4)
Total	5349			

*Abbreviation:* *RCC*, Regional Cancer Centre.

### Disease and treatment data

#### POGONIS

Launched in 1985, POGONIS collects detailed demographic, disease, treatment and outcome data on all patients with a malignancy treated at any of Ontario’s five pediatric cancer centres. Trained data managers collect data prospectively. Data on 1,030 cohort members treated at a pediatric centre will be provided by POGONIS.

#### Chart abstraction

No comparable registry exists for AYA treated at adult centres. Clinical data for 4,319 AYA will be obtained through chart abstraction. Trained abstractors with extensive experience in cancer studies will abstract variables through review of hospital and pharmacy records, radiation planning records, operative and pathology reports, and discharge summaries. Abstractors will work on-site using ICES-developed software that allows entry of personal health information onto encrypted laptops. Abstractors have already received extensive training from the study team including in-depth review of abstraction manuals and mock chart abstraction. A robust protocol for real-time data review will ensure quality abstraction: abstractors will have timely access to study team members for content-related questions. Investigators will review summaries of abstracted charts on a regular basis to ensure data validity, completeness and consistency between abstractors.

#### Data variables

Malignancy-level data will include histology, stage, primary tumour site, laterality, metastatic sites, diagnostic method, and disease status (relapse, progression) (Table 2). We will classify disease using the International Classification of Childhood Cancer, 3rd edition and International Classification of Disease-O systems, allowing comparison of patients treated as these classification systems evolved. Pathology and cytogenetic reports are being scanned to facilitate centralized verification of findings. Total dose (per m^2^) will be calculated for chemotherapies most associated with late effects (e.g. anthracyclines, alkylating agents). Information about clinical trial enrollment and treatment protocols will enable evaluation of the impact of trial enrollment or treatment according to published protocols on survival.

**Table 2:** **Selection of data elements contained in POGONIS and being collected via chart abstraction for AYA treated at adults centres**

Type	Elements
Demographic	Age at diagnosis
	Sex
Treatment plan	Initiation/completion dates
	Protocol names
	Clinical trial enrollment
Diagnosis	Method of diagnosis
	Primary site, laterality
	Stage, staging system
	Extent/size of primary tumour
	Regional lymph node involvement
	Metastases at diagnosis
	Histology, tumour grade
	Molecular markers
Chemotherapy	Plan name
	Chemotherapeutic/biologic agents
	Cumulative doses (mg/m^2^) - selected agents (e.g. anthracyclines, alkylators)
	Dose Units
	Dose Route
Radiation therapy	Intent (curative vs. palliative)
	Start/stop dates
	Radiation site
	Boost site
	Dose
	Fraction number
	Radiation type/technique
Surgery	Date
	Indication/procedure name
	Site
	Margins at resection
	Lymphadenectomy
	Completeness of resection
Hematopoietic stem cell transplantation	Allogeneic vs. autologous
	Source of cells (marrow, peripheral blood stem cells, cord)
	Donor
Outcomes	Relapse (date, sites)
	Progression (date)
	Second malignant neoplasms (date/site)
	Death/last follow up (date, location, cause of death)

### Health services and demographic data

#### Health services data

The cohort will be linked to administrative databases maintained at ICES using a patient-specific encrypted identifier. The Canadian Institute for Health Information (CIHI) Discharge Abstract Database (DAD) contains one record for each hospital stay in Ontario since 1988. The CIHI National Ambulatory Care Reporting System (NACRS) captures information on outpatient visits to hospitals and community-based ambulatory care since 2002. The Home Care Database captures all services provided or coordinated by Ontario’s Community Care Access Centres since 2005. Ontario Health Insurance Plan (OHIP) data contains inpatient and outpatient service claims and procedure billing information paid to physicians, groups, laboratories, and out-of-province providers; healthcare utilization before, during and after treatment can therefore be assessed. Characteristics of physicians involved in the care of cohort members will be determined through the ICES Physicians Database, which includes information on physician specialty, demographics, practice type and location.

#### Patient demographics

These data will be obtained from the Registered Persons Database (RPDB), a vital statistics registry created in 1990 comprised of all individuals who have ever been insured to receive a health service in Ontario. Postal code at diagnosis will be used to determine geographic location (to calculate the shortest distance to a cancer centre), rurality and socioeconomic status (SES) by linkage to census data on median neighbourhood household income (an ecologic measure of SES routinely used in Canadian research).

### Outcome data

Fact of and cause of death will be identified by linkage to the RPDB, and subsequent malignancies will be identified by linkage to the OCR. Both death and subsequent malignancies will be confirmed by chart abstraction using the same methods as the primary malignancy.

### Locus of care

LOC will be categorized into three broad groups based on the primary location of cancer treatment: pediatric centre, RCC, or community hospital. In some circumstances, AYA receive elements of care in different centres. We will define the site of cancer-directed surgery, chemotherapy, radiation and stem cell transplantation for each patient. For AYA who received chemotherapy, LOC will be designated as the site at which the chemotherapy was administered, regardless of other therapies received. For those treated with cancer-directed surgery with and without radiation (but without chemotherapy), LOC will be considered the site of surgery. For evaluation of specific treatments (e.g. intensity of chemotherapy, type of surgery), analyses will be conducted based on the actual location of the specific treatment.

Because surveillance for late effects and palliative care occur after diagnosis, we will redefine LOC for these outcomes. We will determine survivors’ patterns of visits and classify follow-up care in each year hierarchically as cancer-centre/oncologist-based, primary care based, or none.

### Aim 1: To determine the patient and healthcare system factors that determine LOC

We will evaluate the relationship between LOC and type of PCP, distance from RCC and SES.

#### Potential explanatory variables

Patients will be designated as having a pediatrician, a family physician/general practitioner or no PCP in the 2 years prior to diagnosis based on pre-diagnosis healthcare billings. Postal code at diagnosis will be used to geocode patients, hospitals and cancer centres to a geographic point on a spatial areal map. For each AYA, the straight-line distance to the nearest pediatric centre or RCC during the year of the cancer diagnosis will be calculated using a Statistics Canada algorithm [50, 51]. We will use the straight-line distance as a proxy for actual travel burden [52]. SES will be determined by linkage to census data on median neighbourhood household income.

#### Data analysis

We will generate descriptive statistics overall, by PCP type, SES and geographic distance from a pediatric centre or RCC. We will create a multivariate multinomial logistic regression model with PCP type, geographic distance and SES quintile as independent variables, and LOC as the dependent variable. As distance will be positively skewed, we will evaluate the impact of distance as a continuous variable and as a categorical variable in 25 km increments. We will use a general estimating equations approach to adjust for clustering at the PCP level. We will test for interactions between PCP type, geographic distance and SES, and between time period and geographic distance. Of note, we may find patients living far from any treating institution have different patterns of referral than those living closer. To evaluate this, we will identify patients living in communities considered both Northern and Rural by the Ontario Ministry of Health and Long Term Care [53] and will create a separate geographic category for these geographically isolated patients.

### Aim 2: To evaluate the relationship between LOC and AYA care across the cancer continuum

We will evaluate the influence of LOC at three distinct points on the cancer continuum: 1) during active cancer therapy with curative intent; 2) after completion of active treatment; and 3) at end-of-life (if applicable).

#### Cancer therapy

We will limit this analysis to patients undergoing treatment with curative intent, as determined by chart review. Clinical trial enrollment (yes/no) and protocol name will be abstracted. Treatment intensity (chemotherapy, radiation therapy and surgery) will be defined from chart review data for specific malignancies (Table 2). For example, we will evaluate the length of primary therapy, cumulative dose of anthracyclines and use of cranial radiation therapy in patients with acute lymphoblastic leukemia, and the dose and fields of radiation and doses of anthracyclines and alkylating agents in patients with HL.

#### Risk-based survivor care

Based on chart review data, cohort members in remission ≥5 years from diagnosis will be designated as survivors. Using the Children’s Oncology Group guidelines [54], we will identify those survivors at high risk for cardiac dysfunction, secondary breast cancer and colorectal cancer (late effects that cause morbidity and potential mortality, have established surveillance protocols, and are reliably detected using administrative data) (Table 3). Using OHIP billing codes, we will determine adherence to recommended surveillance over time.

**Table 3:** **Definition of risk and required surveillance in survivors at HIGH risk of a late effect**

	Breast cancer	Colorectal cancer	Cardiomyopathy
**Definition of high risk group**	Female, ≥20 Gy radiation therapy to the chest	≥30 Gy radiation therapy to the abdomen, pelvis or spine	Anthracycline +/− chest radiation
**Children’s Oncology Group recommended surveillance for survivors at high risk**	Annual mammogram/MRI beginning 8 years after radiation or age 25 years, whichever occurs last	Colonoscopy every 5 years beginning at age 35 years	Echocardiogram or MUGA
			Annually if anthracycline ≥300 mg/m^2^
			q 2 years if anthracycline 200–300 mg/m^2^ OR anthracycline <300 mg/m^2^ + radiation q 5 years if anthracycline <200 mg/m^2^, no radiation

#### End-of-life palliation

For this analysis, the study population will consist of cohort members who died after experiencing relapse or progression, thereby excluding deaths related to toxicity of initial cancer therapy. Access to formal palliative care services will be determined through inpatient and outpatient palliative care claims.

#### Data analysis

A variety of statistical methods will be used. Multivariable logistic regression modeling will be implemented to examine factors associated with being enrolled on a clinical trial and intensity of cancer therapies. The main exposure will be LOC and the model will be adjusted for demographic, disease, and provider characteristics. A generalized estimating equations approach [55] will be used to account for clustering of patients within individual centres. Relationships between covariates will be explored using the variance influence factor to ensure that highly correlated variables are not included together in multivariable regression models. If two variables are highly correlated, we will include the variable that is deemed most clinically relevant to the outcome.

To evaluate repeated events (e.g. risk-based survivor care), we will examine the relationship of LOC with breast imaging, echocardiogram/MUGA, and colorectal cancer screening in those survivors at high risk for specific late effects. The timing of these repeated events varies depending on the care recommended in the guidelines. Using a counting process model (based on a Poisson process) [56], the rate of event occurrence for each patient will be modeled as a function of time, and available covariates. The model will incorporate fixed and time-dependent covariates (LOC for follow-up care may change yearly). Likelihood-based methods will be used to estimate the regression parameters [57].

We will use time to event methods to evaluate the relationship between LOC and end-of-life palliative care. Timing of palliative care in relation to major events (diagnosis, relapse/progression, death) will be described. Cox proportional hazards regression will model the association of LOC with time to palliative care involvement, using the first relapse/progression as time zero. Subsequent relapses/progressions will be treated as time-varying covariates. Variable interactions with time period will be explored.

Given our robust chart review process, and because most outcomes will be determined through linkage to administrative data, we expect few data to be missing. However, to handle missing data for a specific variable, we will first assess whether the data are missing completely at random (MCAR), missing at random, or missing not at random [58]. If the data are MCAR, then we will proceed with complete case analysis. Although there is a loss of power with this approach, the estimated regression parameters are not biased by the absence of the data. When data are not MCAR, multiple imputation methods will be implemented; these techniques produce unbiased parameter estimates and provide adequate results in the presence of low sample size or high rates of missing data [58].

### Aim 3: To examine the relationship between LOC and survival within malignancy groups accounting for potential confounders

We will evaluate overall (OS) and event-free survival (EFS) by LOC for a variety of tumor groups including, leukemia, NHL, soft tissue/bone sarcomas and brain tumours.

#### Treatment

Chemotherapy intensity will be assessed by examining four categorical scores, one for each of alkylating agents, anthracyclines, epipodophyllotoxins and platinum agents. For anthracyclines, all cumulative does will be converted to doxorubicin equivalents. Similarly, alkylating agents will converted to cyclophosphamide equivalent doses [59]. Radiotherapy will be assessed by a yes/no categorical variable along with summarized information of the total dose received for each anatomical field. Hematopoietic stem cell transplant will be assessed by a 4-level categorical variable (0 = no transplant, 1 = autologous transplant, 2 = allogeneic transplant, related donor and 3 = allogeneic transplant, un-related donor). Surgery will be assessed by a yes/no categorical variable along with summarized information of the extent of resection. We will also document the duration of the primary therapy. Some treatment modalities will not be applicable to all diagnostic groups. Clinical trial enrollment will be included as a dichotomous variable.

#### Covariates

Age (by year, and categorized as adolescent vs. adult), sex (when applicable) and SES (quintiles) will be determined from the RPBD. Other disease factors such as stage, grade and molecular markers will be included were appropriate (e.g. Philadelphia chromosome in ALL).

#### Data analysis

We will report crude rates of OS and EFS for the entire cohort and for each disease group, by LOC. To study OS, standard techniques for survival analysis will be applied. For each malignancy group, the Kaplan-Meier approach will be used to obtain a non-parametric estimate of the survivor function for each LOC, separately. The Nelson-Aalen approach will be used to provide nonparametric estimates of the cumulative hazard functions. Similar techniques will be applied to study EFS.

To model OS for the entire cohort and for each malignancy, we will use a Cox proportional hazards regression approach to examine the relationship between OS and pre-specified covariates of key interest (above). Multiple other variables that may influence cancer survival will be available through chart review and administrative data; analyses that include these factors will be considered exploratory. We will conduct several tests, including examining residual plots, to ensure the proportional hazards assumption is appropriate. If violated, we will expand the model by exploring various interactions between time and the covariate in question. Centre-specific random effects [60] will be incorporated into Cox regression models to account for correlation that may arise due to clustering of patients within centres. To model EFS for the entire cohort and for each malignancy, we will use similar techniques as discussed for OS. Missing data will be treated as described in Aim 2.

Our cohort spans diagnoses identified over a 20-year period. As such, cohort effects would normally be considered in the analysis phase. Previous work examining the effect of LOC has indeed used period of diagnosis as a prognostic factor and in all studies, it was used as a proxy for differences in diagnostic techniques and treatment approach. Given that the central aim of our analysis is to examine differences in diagnosis and treatment, and that we will have collected detailed data on all treatment exposures, period of diagnosis will not be relied upon as a proxy for these exposures.

### Sample size and power considerations

We have provided power calculations for selected hypotheses across aims 1–3. These hypotheses were selected to highlight power sufficiency even for hypotheses with limited sample sizes.

#### Aim 1

To assess the hypothesis that increasing distance from a pediatric centre or RCC will be associated with a lower likelihood of referral to a cancer centre (pediatric centre or RCC) versus a community hospital, the total cohort of 5,349 patients will utilized. It is estimated that approximately two thirds live less than 50 km away from a cancer centre. Assuming that the average probability of attending a cancer centre is 76% (4,065/5,349), there will be 80% power to detect at least a 4% absolute difference in probability of attending a cancer centre between patients living less than 50 km away from a cancer centre vs. those living greater than 50 km away. These calculations use a two-sided binomial test with alpha of 0.05.

#### Aim 2

We present the power to demonstrate important differences in clinical trial enrollment and palliative care. Of the 5,349 patients in our cohort, 1,030 patients attended a pediatric centre and 3,035 patients attended a RCC. From POGONIS, we know that the average probability of being enrolled on a clinical trial among those AYA treated in a pediatric centre is 54%. Therefore, there will be 80% power to detect at least a 5.1% absolute difference in trial enrollment rates between patients attending a pediatric centre and those attending a RCC. These calculations use a two-sided binomial test with alpha of 0.05.To assess the hypothesis that end-of-life care is associated with LOC, we will only examine disease-related deaths. Of the 931 deaths identified in our cohort, we estimate that 740 (80%) are disease-related. We estimate that approximately one third of patients dying of cancer will receive palliative care. We also estimate that 584 of the 740 patients will have been treated in a cancer centre (pediatric or RCC) at the time of their last cancer treatment and 156 at community hospitals. Assuming that 60% of terminal AYA treated at a cancer centre receive palliative care services within 2 months of their death, we will have 80% power to detect at least a 35% higher rate of receiving palliative care among patients in cancer centres versus patients in community hospitals. These calculations are based on the log rank test, type I error alpha 0.05.

#### Aim 3

Survival probabilities for patients treated in a pediatric centre have been provided by the Pediatric Oncology Group of Ontario. For sarcomas (152 pediatric; 273 RCC), based on a 5-year mortality rate of 32% in a pediatric centre, we will have 80% power to detect at least a 44% increase in hazard of death for patients treated in an RCC vs. pediatric centre. For leukemia (184 pediatric; 255 RCC), based on a 5-year mortality rate of 21% among patients treated in a pediatric centre, we will have 80% power to detect at least a 49% increase in hazard of death for patients treated in an RCC vs. pediatric centre. For brain tumours (172 pediatric; 221 RCC), based on a 5-year mortality rate of 31% among patients treated in a pediatric centre, we will have 80% power to detect at least a 46% increase in hazard of death for patients treated in a RCC vs. pediatric centre. For NHL (109 pediatric; 212 RCC), based on a 5-year mortality rate of 25% among patients treated in a pediatric centre, we will have 80% power to detect at least a 59% increase in hazard of death for patients treated in a RCC vs. pediatric centre. The power calculation uses a log rank test with type I error alpha of 0.05.

## Discussion

Cancer is the leading cause of disease-related death in AYA, yet healthcare systems frequently fail to meet the needs of this vulnerable group [21]. Critical outcomes such as improvement in survival over time and access to supportive care have not kept pace with those in children or the elderly. Given the many life years impacted by a cancer diagnosis for AYA, these deficiencies in care must be addressed. AYA aged 15–21 may be treated in a specialized pediatric oncology unit within a pediatric centre, at an RCC, or a community hospital. Consequently, these young people are most likely to benefit from research exploring the relationship between LOC and the types and intensity of treatment, access to clinical trials, palliative and survivor care, and most importantly, their chance of survival. An increased focus by government and the cancer community [61–65] on disparities in AYA cancer care has created an opportunity to effect change in provincial and national cancer policy that will determine where AYA are treated and what medical and supportive care resources are essential to optimize care. Cancer Care Ontario and other provincial cancer agencies have launched initiatives to create specialized “Service Provider Sites” for individual malignancy groups (e.g. sarcoma) to ensure equitable access to high quality cancer services with integrated, multidisciplinary expertise. Provider sites will be concentrated in a limited number of institutions to ensure sufficient volume to maintain expertise. Our analysis will inform similar initiatives focused on ensuring that AYA can access quality cancer care.

Beyond influencing policy regarding the optimal LOC for AYA that will ensure equal opportunity for survival, the work performed using this cohort will impact the care of AYA with terminal cancer and those who become long-term survivors. Data regarding access to appropriate palliative care for AYA is sparse. This study will provide foundational information by identifying factors that impede prompt AYA access to palliative care. It will inform targeted policies to ensure all AYA with terminal cancer receive early and appropriate palliative care (such as immediate introduction of palliative care to at-risk subgroups). It will also aid in efforts advocating for novel programs and technologies with the potential to improve end-of-life AYA care [66–68]. This analysis will inform potential strategies to improve AYA survivor care: the creation of dedicated AYA survivor programs in a limited number of centres, expansion of existing programs for pediatric cancer survivors, or education initiatives to improve survivor and PCP knowledge and compliance with surveillance guidelines.

Beyond its impact on guiding policy, the IMPACT cohort will provide an unparalleled resource for future research. The IMPACT data platform will be established as a permanent resource enabling other investigators with an interest in AYA cancer to perform their own investigation into AYA cancer. The level of detail available in the database and via linkage to ICES’ other data holdings will create numerous opportunities to complete studies that address a range of issues that span the AYA cancer journey and have the potential to improve both the quantity and quality of AYA cancer survival.

## Abbreviations

ALL: Acute lymphoblastic leukemia
AYA: Adolescent and young adults
CIHI: Canadian Institutes of Health Information
DAD: Discharge abstract database
EFS: Event-free survival
GEE: Generalized estimating equations
HL: Hodgkin’s lymphoma
ICES: Institute for Clinical Evaluative Sciences
IPDB: ICES physicians database
LOC: Locus of Care
MCAR: Missing completely at random
NACRS: National Ambulatory Care Reporting System
NHL: Non-Hodgkin’s lymphoma
PCP: Primary care practitioner
RCC: Regional cancer centre
RPDB: Registered persons database
OCR: Ontario Caner Registry
OHIP: Ontario Health Insurance Plan
OS: Overall survival
POGONIS: Pediatric Oncology Group of Ontario Networked Information System
SES: Socioeconomic status.
